# In Vitro Fish Cell Culture: From Primary Muscle Cells to Cell-Based Meat in Cyprinidae

**DOI:** 10.3390/biology15030291

**Published:** 2026-02-06

**Authors:** Piyathip Setthawong, Chanati Jantrachotechatchawan, Suppakorn Netmanee, Napat Tandikul, Chaiyaboot Ariyachet, Witchukorn Phuthong, Kornsorn Srikulnath

**Affiliations:** 1 Department of Physiology, Faculty of Veterinary Medicine, Kasetsart University, Bangkok 10900, Thailand; piyathip.s@ku.th; 2 AI-XENIX Co., Ltd., Bangkok 10220, Thailand; 3 Department of Biochemistry, Faculty of Science, Kasetsart University, Bangkok 10900, Thailand; 4 Department of Physics, Faculty of Science, Kasetsart University, Bangkok 10900, Thailand; 5 Department of Biochemistry, Faculty of Medicine, Chulalongkorn University, Bangkok 10330, Thailand; 6 Animal Genomics and Bioresource Research Unit (AGB Research Unit), Faculty of Science, Kasetsart University, Bangkok 10900, Thailand; 7 Department of Genetics, Faculty of Science, Kasetsart University, Bangkok 10900, Thailand; 8 Special Research Unit for Wildlife Genomics (SRUWG), Department of Forest Biology, Faculty of Forestry, Kasetsart University, Bangkok 10900, Thailand; 9 Biodiversity Center, Kasetsart University (BDCKU), Bangkok 10900, Thailand

**Keywords:** Cyprinidae, fish muscle cell, cell culture, cultured meat

## Abstract

Fish offer an excellent source of high-quality protein with balanced nutrients and low fat content. However, the increasing global demand for food and the impacts of climate change have led to a significant decline in wild fish stocks. Cultivated fish meat has therefore emerged as a promising sustainable food alternative. In this review, we summarize the structural and physiological characteristics of fish muscle and highlight the methods used to establish primary muscle cell cultures, including explant outgrowth and enzymatic dissociation, alongside the optimization of environmental conditions and growth media composition. Particular attention is given to the isolation, development, and characterization of Cyprinidae muscle-derived cell lines via morphological assessments, gene expression profiling, and karyotyping. In addition, we discuss recent advances in scaffold-based and three-dimensional culture systems as well as the application of bioreactors for large-scale production. Current challenges include the limited availability of standardized muscle cell lines, dependence on serum-containing media, and the high cost of growth factors. Future progress will depend on innovations in serum-free formulations, cost-effective media, and reproducible culture protocols. Ultimately, fish muscle cell culture supports not only the development of alternative protein sources but also aquatic health research, disease modeling, and sustainable bioproduction.

## Simple Summary

Fish provide high-quality protein and important nutrients, but wild fish stocks are declining because of growing food demand and climate change. Cultivated fish meat, which is grown directly from cells, has therefore emerged as a promising approach to support a sustainable and reliable food supply. This review explains the basic structure of fish muscle and describes how scientists grow muscle cells outside the body. We focus particularly on fish from the Cyprinidae family, which includes many widely farmed species. The review outlines common methods for obtaining muscle cells, such as allowing small pieces of tissue to grow out in a dish or using gentle enzymes to separate individual cells. We also describe the conditions needed for these cells to grow, including suitable temperatures, nutrients, and supportive materials that help the cells form muscle-like structures. Recent progress in three-dimensional growing systems and controlled vessels may enable larger-scale production in the future. However, challenges remain, including the limited number of well-characterized Cyprinidae muscle cell types and the high cost of the nutrients required for cell growth. Continued research will help improve these methods and support sustainable food production and aquaculture.

## 1. Introduction

The global food system, particularly aquaculture, is facing escalating challenges due to the overuse of natural resources and climate change [1]. Overfishing, driven by the growing global demand for seafood, has resulted in the depletion of numerous fish stocks, with over a third of global stocks being fished at biologically unsustainable levels, which is threatening the long-term viability of marine ecosystems [2]. Pollution, including pervasive plastic waste, nutrient-rich agricultural runoff, and toxic industrial discharge, has produced widespread habitat degradation [3,4]. It further causes direct harm to aquatic life through ingestion and entanglement, thereby creating hypoxic “dead zones.” Rising water temperatures, ocean and freshwater acidification, and pollution have collectively disrupted aquatic ecosystems, which has led to a marked decline in fish productivity and biodiversity [5,6,7]. Such stressors have altered species distribution, disrupted food webs, and degraded critical habitats [8]. These combined effects threaten traditional fisheries, not only marine but also entire aquatic species.

Among freshwater fish, the Cyprinidae family is the largest and most diverse, as it encompasses thousands of species distributed across the globe [9]. These fish offer particular promise due to their nutritional value and established significance in aquaculture [10]. Moreover, the Cyprinidae species are economically important across Asia and Europe, as it supports both subsistence and commercial aquaculture systems [11]. Especially in Southeast Asia, the Tor genus or Mahseer fish, which also belong Cyprinidae family, are economically valuable. For example, *Tor soro* are consumed as food and found in lakes, rivers and waterfalls. They live in clean, highly oxygenated and strong currents. They contained high protein, low-fat compositions, macrominerals and microminerals [12]. However, the threatening stressors, such as overfishing, have driven them close to extinction [13]. The exemplification emphasizes alternative sustainable protein sources, balancing the consumption and biodiversity conservation [2,14].

The understanding of the complex fish muscle development process could facilitate addressing these challenges. It is governed by the interplay of two primary mechanisms: hyperplasia, the formation of new muscle fibers, and hypertrophy, the expansion of existing fibers [15,16]. Central to these processes are myosatellite cells, a population of resident muscle stem cells. In response to growth signals or injury, these quiescent cells become activated, proliferate as myoblasts, and subsequently differentiate and fuse to form new multinucleated myotubes or to augment existing muscle fibers [17,18]. This cellular-level activity directly determines the ultimate texture, structure, and yield of the muscle tissue. The dynamics of hyperplasia and hypertrophy vary significantly across fish species and life stages, influencing the final quality of the flesh [19].

Adopting this fundamental knowledge, the current aquaculture practices have demonstrated that the physiological characteristics of fish muscle are highly plastic and can be modulated through targeted interventions. Specifically, nutritional strategies have proven effective in enhancing flesh quality. For instance, the optimization of dietary amino acid profiles, such as the supplementation of leucine or glutamine, can stimulate protein synthesis and muscle growth through signaling pathways like AMPK/Sirt1 [20]. Similarly, the inclusion of specific lipids, vitamins, and minerals in aquafeeds can influence the fatty acid composition, oxidative stability, and overall nutritional value of the muscle [21]. These interventions highlight that a sophisticated understanding of muscle cell physiology allows for the precise control of flesh quality attributes, a principle that is directly relevant to emerging food technologies.

The level of control is becoming increasingly important as contemporary expectations for meat quality are more rigorous than ever. To predict the approaching trend, modern consumers will demand not only a safe and affordable product but also one that delivers a superior sensory experience and a robust nutritional profile [22]. Key quality attributes such as texture (firmness, juiciness, and tenderness), flavor, color, and water-holding capacity are now critical determinants of market success. The demand for high-quality protein, rich in essential amino acids and beneficial lipids like omega-3 fatty acids, further underscores the need for a deep understanding of the biological factors that shape these characteristics [21,23].

Cultivated fish meat, which is produced through the in vitro culture of fish muscle cells, represents an emerging solution consistent with the principles of sustainability, food security, and animal welfare [24,25]. The successful development of cell-based fish meat that meets high consumer expectations is contingent upon recapitulating the complex biological processes of myogenesis in a controlled environment. The established knowledge of how nutrient interventions modulate muscle cell physiology and flesh quality in conventional aquaculture provides an invaluable roadmap for such an endeavor. By applying these principles, it is possible to guide the proliferation and differentiation of myosatellite cells in vitro to create structured, nutritious, and palatable muscle tissue. While terrestrial cell-cultivated meat development can draw upon a robust body of biomedical tissue-engineering research and a comparatively comprehensive knowledge base in mammalian and avian cell biology, the scientific foundations supporting cell-cultivated fish remain considerably less mature, resulting in a longer route toward large-scale production [24]. Harnessing the proliferative and myogenic potential of these satellite cells in vitro thus forms the fundamental basis of the production of cell-based or cultivated meat [26].

Despite substantial progress in mammalian cell culture and cultivated meat research, the application of similar techniques to aquatic species remains underdeveloped. Major limitations include the absence of standardized protocols for primary cell isolation, serum-free growth media formulations, and scalable culture systems [27,28]. Addressing these issues requires a detailed understanding of fish muscle physiology, cellular differentiation, and the environmental parameters that are optimal for growth and maintenance [29]. The development of cell-based fish meat is occurring within an evolving global regulatory framework. Agencies such as the U.S. Food and Drug Administration and the European Food Safety Authority are establishing guidelines to assess the safety and labeling of these novel food products, as these factors will be critical for their commercialization and to secure public trust [30].

In this review, we examine the current state of knowledge on Cyprinidae muscle cell culture, with an emphasis on methodological approaches, cell line characterization, and technological innovations, such as scaffolds, three-dimensional systems, and bioreactors. We further highlight existing technical and economic constraints and identify research priorities to advance cell-based aquatic foods as a viable component of sustainable aquaculture and the future food system.

## 2. Methods

To provide an overview of cyprinid cell culture studies, a comprehensive literature search was conducted using the Scopus database. Representative publications were selected for in-depth analysis of methodologies related to cyprinid cell isolation, in vitro culture conditions, and subsequent characterization protocols (Table 1). Cyprinid fish cell lines are most commonly derived from fin tissue, which predominantly yields fibroblast-like cells [29]. Although fibroblasts support extracellular matrix (ECM) production, an excess can alter tissue composition and reduce muscle yield, which is a key consideration for cell-based meat production. Strategies to enhance myogenic purity include pre-plating, optimized enzymatic dissociation, and supplementation with myogenic growth factors (e.g., bFGF) under reduced serum conditions [25]. Implementing these approaches is essential for advancing cyprinid muscle cultures toward scalable and reproducible cell-based meat applications [27]. To support methodological comparisons, Table 2 summarizes key operational parameters critical for successful muscle cell isolation and early-stage culture in Cyprinidae species, as reported in the original studies. Additionally, Table 3 presents a summary of Cyprinidae fish cell lines registered in the Cellosaurus database.

**Table 1:** Summary of the Cyprinidae fish cell lines.

Species	Original Cell Source	Morphology	Medium	Method	Incubation	Passages	Chromosome Analysis	Characterization	Reference
*Tor putitora* (Ham)	1. Muscle 2. Fin	Fibroblast-like morphology	- L-15 with 20% FBS and 10% fish muscle extract (FME) - After 10 passages: L-15 with 10% FBS	Enzyme digest: 0.2% trypsin until the fluid became turbid	28 °C	More than 20	100 chromosomes at passages 10 and 20	- [^3^H]-thymidine uptake assay to measure growth - Cell cycle analysis by fluorescence-activated cell sorting (FACS) revealed that most of the cells on the first and fourth day of culture were in S-phase, indicating a high growth rate	[31]
*Carassius auratus* (Goldfish)	1. Muscle 2. Swim bladder	1. Muscle cells were epithelial in primary culture, with spider-like projections 2. Swim bladder cells were fibroblastic from the initial to the current passage	L-15 with 20% FBS + 1% antibiotics (100 µg/mL penicillin, 100 IU/mL streptomycin) + 25 ng/mL epidermal growth factor (EGF) + 25 ng/mL bFGF	Explant technique: 25 tissue fragments (1–2 mm^3^) were individually explanted into 25 cm^2^ tissue culture flasks.	30 °C	More than 35	104 chromosomes	- Ribosomal RNA analysis - Viral susceptibility test	[32]
*Cyprinus carpio* (common carp)	1. Fin 2. Heart	- Cell migration observed at 2–3 days - Fin cells were fibroblasts and epithelial cells during initial growth, and changed to epithelial-like cells - Heart cells were fibroblasts and myocytes in primary culture	L-15 with 10% FBS + bFGF 10 ng/mL	Explant technique: 25 tissue fragments (1–2 mm^3^) were individually explanted into 25 cm^2^ tissue culture flasks.	28 °C	1. Fin: 49 2. Heart: 51	1. Fin: varied from 46 to 121 2. Heart: varied from 47 to 126	Cell lines originated from common carp, amplifications of 551 and 655 bp fragments of 16S rRNA and COI gene sequences	[33]
*Tor tor* (Tor mahseer)	1. Fin 2. Heart	- Cell migration observed at 3 days 1. Fin: Heterogeneous cell layer (epithelial and fibroblastic cells) around fin after 7–10 days 2. Heart: Homogeneous layer of fibroblastic cells (a sharp and clear outline) from heart cells after 12–13 days	L-15 with 20% FBS + antibiotics (500 IU/mL penicillin and 500 µg/mL streptomycin and 2.5 mg/mL fungizone) at pH 7.4	Explant technique: Tissues were minced into small pieces and seeded into cell culture flasks and allowed to attach to the surface.	28 °C	1. Fin: 15 2. Heart: 13	-	Phase-contrast photomicrographs	[34]
*Labeo rohita* (Rohu)	1. Fin 2. Heart 3. Swim bladder	- Both epithelial and fibroblast-like cells in initial subcultures of fin and swim bladder cell lines - Fibroblast-like cells and myocytes in the heart cell line	- Initial 10 passages: L-15 with 15% FBS (Invitrogen, Carlsbad, CA, USA) - After 10 passages: L-15 with 10% FBS (Invitrogen, Carlsbad, CA, USA)	Explant technique: 25 tissue fragments (1–2 mm^3^) were individually explanted into 25 cm^2^ tissue culture flasks.	28 °C	1. Fin: 50 2. Heart: 55 3. Swim bladder: 51	50 chromosomes	The amplification of 496 and 655 bp fragments of 16S rRNA and cytochrome oxidase subunit I of mtDNA	[35]
*Puntius denisonii* (Miss Kerala)	1. Fin 2. Heart	- Cell migration observed at 4–5 days	- Initial 10 passages: L-15 with 15% FBS (Invitrogen, Carlsbad, CA, USA) - After 10 passages: L-15 with 10% FBS (Invitrogen, Carlsbad, CA, USA)	Explant technique: 25 tissue fragments (1–2 mm^3^) were individually explanted into 25 cm^2^ tissue culture flasks.	26 °C	1. Fin: 60 2. Heart: 55	50 chromosomes at passages 50	- Phase-contrast photomicrographs - The amplification of 653 bp fragments of cytochrome oxidase subunit I of mitochondrial DNA genes.	[36]
*Puntius sophore* (Hamilton)	Caudal fin	- Cell migration observed at 2–3 days. - Confluent monolayer in 5–7 days. - Both epithelial and fibroblast-like cells in the initial subcultures of the cell line - In later cultures, predominance of fibroblast cells only	- Initial 10 passages: L-15 with 15% FBS (Invitrogen, Carlsbad, CA, USA) - After 10 passages: L-15 with 10% FBS (Invitrogen, Carlsbad, CA, USA)	Explant technique: 25 tissue fragments (1–2 mm^3^) were individually explanted into 25 cm^2^ tissue culture flasks.	28 °C	104 over a period of 1.5 years	- 50 chromosomes at 25 and 50 passages - 52 chromosomes at 70, 85, and 100 passages	- Population doubling time of 25 h - Immunocytochemical staining was positive for vimentin and negative for cytokeratin. - The origin of the cell lines was confirmed by the amplification of 581 and 655 bp fragments of 16S rRNA. - Microsatellite analysis	[37]
*Puntius (Tor) chelynoides*	1. Eye 2. Fin 3. Heart 4. Swim bladder	- Cell migration observed at 2–3 days - Eye: epithelial and fibroblast-like cells - Fin and heart: epithelial and fibroblast-like cells - Swim bladder: fibroblast-like cells	L-15 with 20% FBS	Explant technique: 25 tissue fragments (1–2 mm^3^) were individually explanted into 25 cm^2^ tissue culture flasks.	24 °C	1. Eye: 31 2. Fin: 15 3. Heart: 9 4. Swim bladder: 7	100 chromosomes	Amplification of mitochondrial cytochrome oxidase subunit I (COI) and 16S rRNA genes	[38]
*Anabarilius grahami*	Caudal fin	- Cell migration at 5–6 days - Fibroblast-like morphology - Monolayers of primary cells after 30–40 days	DMEM/F12 with 20% FBS (Gibco, Grand Island, NY, USA) + antibiotics (100 IU/mL penicillin, 100 mg/mL streptomycin)	Explant technique: minced into about 1 mm^2^ and transferred into T-25 flasks	28 °C	More than 60	48 chromosomes	Cell origin identification by chromosome analysis	[39]
*Tor Tor*	Caudal fin	- Cell migration observed at 3–4 days - Both fibroblast- and epithelial-like cells during the initial subcultures - Predominantly fibroblast cells after 7 passages	L-15 with 20% FBS (Invitrogen, Carlsbad, CA, USA) + bFGF 10 ng/mL	Explant technique: 25 tissue fragments (1–2 mm^3^) were individually explanted into 25 cm^2^ tissue culture flasks.	28 °C	64 over a period of 1.5 years	100 chromosomes at passages 15, 30, 45 and 60	- Immunocytochemical staining showed positive for vimentin and negative for cytokeratin. - The origin of the cell lines was confirmed by the amplification of 578 and 655 bp sequences of 16S rRNA and cytochrome oxidase subunit I genes of mitochondrial DNA.	[40]
*Danio* *rerio* (Zebrafish)	Muscle	Spindle-shaped fibroblast cells	L-15 with 10% FBS (Invitrogen, Carlsbad, CA, USA) + bFGF 10 ng/mL without the use of antibiotics	Explant technique: chopped into small pieces (1 mm^3^)	28 °C	61 over a period of 12 months	-	- DNA barcoding (16S rRNA and COX1) was used to authenticate the cell line. - Immunocytochemistry: positive staining to vimentin	[41]
*Danio* *rerio* (Zebrafish)	Muscle	- Formation of early myotube after 3–4 days in differentiation medium - Late-stage: myotubes	L-15 with 0.8 mM CaCl_2_, 2 mM glutamine, 20% FBS, 100 µg/mL penicillin/streptomycin	Enzyme digest: 5 mg/mL collagenase type 1 at room temperature for 45 min in a shaker at 200 rpm	28 °C	-	-	- Differentiation to fuse and form multinucleated myotubes - Desmin positive, which reflected their muscle specificity - mRNA expression of the muscle regulatory factors MyoD, myogenin, and Myf6	[42]
*Pimephales promelas* (fathead minnow)	Muscle	-	M199 with 10% FBS	-	27 °C with 2% CO_2_ in 98% air	4–5	-	- Sequence analysis and phylogenetic analysis - Gene expression analysis by real-time quantitative RT-PCR (qRT-PCR)	[43]
*Carassius auratus* (grass goldfish)	Muscle	- Migration of mononuclear muscle cells began from the explants after day 5 - Long, spindle-like and multipolar-like cells	L-15 with 15% FBS (Gibco, Grand Island, NY, USA) and 10% fish muscle extract	Enzyme digest and explant: 0.25% trypsin solution digested at room temperature for 20 min and tissue explant	23 °C	More than 89	150 chromosomes	- Chromosome analysis and COI gene analysis	[44]
*Labeo rohita*	Muscle	Activated satellite cells or myoblasts	- Initial 5 passages: L-15 with 10% FBS + 10 ng/mL bFGF - After 5 passages: L-15 with 15% FBS + 10 ng/mL bFGF	Explant technique: Muscle tissue explanted into a flask. Cell radiation from the explant started after 120 h. The first subculture was after 12 days.	28 °C pH 7.4	More than 34	-	- Authentication: mitochondrial 16S rRNA sequencing analysis - qRT-PCR: Mrf4, MyoD, MyoG, Myf5, MEF2A, and ꞵ-actin - Transfection capacity: pmaxGFP by Lipofectamine 3000^®^ - Susceptibility: extracellular products of *Aeromonas hydrophilla* and *Edwardsiella tarda*	[45]
*Carassius carassius*	Gill cell	Cell migration with a fibrillar morphology was observed at 2 days	- Initial 8 passages: M199 with 20% FBS (Gibco, Grand Island, NY, USA) + antibiotics (200 IU/mL penicillin, 200 mg/mL streptomycin, 50 mg/mL gentamicin, and 5 mg/mL amphotericin B) - After 8 passages: M199 with 15% FBS (Gibco, Grand Island, NY, USA)	Enzyme digest: 0.25% trypsin at room temperature for 25 min	25 °C	90	46 (ranged from 18 to 68) at passage 50	Species authentication: The 16S rRNA analysis confirmed that the CCG cell line was exclusively derived from Carassius auratus, with a 98% sequence match.	[46]

**Table 2:** Key Operational Parameters for Muscle Cell Isolation and Culture in Cyprinidae.

Species	Enzymatic Dissociation (Enzyme, Concentration, Time, Temperature)	Explant Method (Tissue Size, Incubation)	Culture Conditions (Medium, FBS %)	Temperature	Reported Outcome	References
*Carassius auratus* (Goldfish)	Trypsin, 0.25%, 20 min, room temperature	Explant fragments (1–2 mm^3^)	L-15 or DMEM supplemented with 15–20% FBS (10% FME, bFGF or EGF)	23 °C, 30 °C	Robustly proliferating muscle-derived cell lines with fibroblast-like morphology.	[32,44]
*Danio rerio* (Zebrafish)	Collagenase type I, 5 mg/mL, 45 min, room temperature	Minced tissue (~1 mm^3^)	L-15 supplemented with 10–20% FBS (bFGF)	28 °C	Myogenic muscle cell line capable of differentiation and formation of aligned myotubes, including in three-dimensional collagen constructs.	[41,42]
*Labeo rohita*	Not reported	Minced tissue	L-15 supplemented with 15–20% FBS (bFGF)	28 °C	Muscle-derived cell line exhibiting stable proliferation and expression of myogenic markers, suitable for in vitro functional studies.	[45]
*Tor putitora*	Trypsin, 0.2%, until tissue dissociation	Minced tissue	L-15 supplemented with 20% FBS (10% FME), later reduced to 10% FBS	28 °C	Fibroblast-like cell line exhibiting stable proliferation, primarily applied to general cell biology and aquaculture research.	[31]

**Table 3:** Summary of Cyprinidae fish cell lines registered in Cellosaurus.

Name	Synonym/Description	Cellosaurus Accession	Species	Comments	Reference
CCF-K104	Carp Caudal Fin-Kochi 104	CVCL_X184	*Cyprinus carpio* (Common carp)	Optimal growth: 25–30 °C	[47]
FtGF	Fantail Goldfish Fin	CVCL_A8S3	*Carassius auratus* (Fantail goldfish)	Doubling time: 33.9 h at 25th passage	[48]
RyuF-2	Ryukin Fin-2	CVCL_UC76	*Carassius auratus* (Ryukin goldfish)		[49]
GiCB	Gibel Carp Brain	CVCL_CW64	*Carassius gibelio* (Prussian carp) Alias: *Cyprinus gibelio*		[50]
EPC	Epithelioma Papulosum Cyprini	CVCL_4361	*Pimephales promelas* (Fathead minnow) Family: Leuciscidae	Doubling time: ~36 h (American Type Culture Collection: CRL-2872) Cell type: Epithelial cell	[51]
KF1	Koi Fin-1; KF-1	CVCL_W095	*Cyprinus carpio* (Koi common carp)	Cell type: Fibroblast	[52]
CCB	Common Carp Brain; Cyprinus Carpio Brain	CVCL_W096	*Cyprinus carpio* (Common carp)		[53]
CCKF	KCF; Cyprinus Carpio Koi Fin	CVCL_1G73	*Cyprinus carpio* (Koi common carp)		[54]
CTE	Catla Thymus Epithelial	CVCL_R826	*Labeo catla* (Catla) Alias: *Catla catla*	Cell type: Thymic epithelial cell	[55]
CTM	Catla Thymus Macrophage	CVCL_1G80	*Labeo catla* (Catla) Alias: *Catla catla*	Cell type: Thymic macrophage	[56]
FHMT-W1	FHMT; Fat Head Minnow Testis	CVCL_L018	*Pimephales promelas* (Fathead minnow) Family: Leuciscidae		[57]
CIK	Ctenopharyngodon Idellus Kidney	CVCL_CV32	*Ctenopharyngodon idella* (Grass carp) Family: Xenocyprididae		[58]
CFS (Carassius)	Carassius Fin from Lake Suwa	CVCL_6F75	*Carassius langsdorfii* (Japanese silver, Ginbuna crucian carp)		[59]

## 3. Muscle Fiber Organization and Function in Cyprinidae Fish

Skeletal muscle in fish constitutes the primary edible portion, and its structural characteristics influence both the locomotor performance and nutrient composition of fish. Muscle growth occurs through hypertrophy (an increase in myofiber size) and hyperplasia (an increase in myofiber numbers). Fish skeletal muscles are generally classified into three fiber types based on their morphology and metabolic properties: white, red, and pink fibers [60].

White fibers are fast-twitch, anaerobic, and highly glycolytic and support short-duration, high-intensity swimming. Due to limited vascularization, white fibers rely primarily on anaerobic glycolysis for rapid ATP production. In contrast, red fibers are slow-twitch, highly aerobic, and located as a triangular band along the lateral line [61]. Extensively vascularized and characterized by dense capillary networks that support their high oxygen demands, red fibers help fish maintain sustained, moderate-speed swimming. These fibers, which are rich in myoglobin, mitochondria, glycogen, and lipids, typically account for less than 10% of total skeletal muscle. Meanwhile, pink fibers represent an intermediate type: they combine fast contractions with a moderate aerobic capacity to provide a functional and morphological transition between the white and red fibers [61,62].

**Figure 1:**
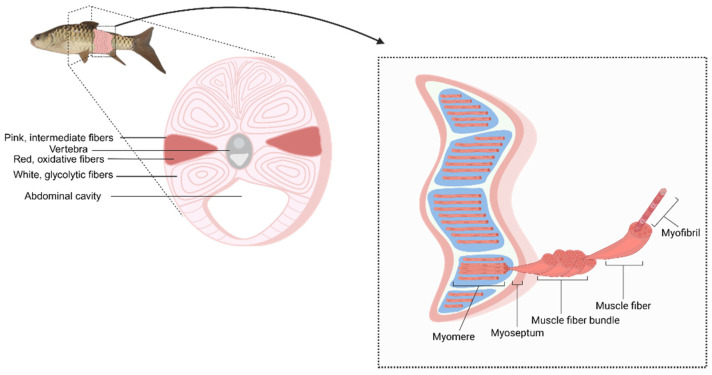
Schematic representation of the hierarchical structure of vertebrate muscles represented by the muscle of a Cyprinid fish cut longitudinally and in cross-section to show a cross-sectional view of a muscle with a single fascicle, muscle fiber, and myofibril. The single muscle is oriented such that the muscle fibers run parallel to the page.

Similarly to all other vertebrates, the muscle fiber microstructure of fish comprises myosin, which is composed of two heavy chains and four light chains. Fast-twitch (white) muscle fibers and pink fibers, which have intermediate contraction speeds, display an identical light chain myosin composition. White muscle fibers, which express fast-twitch myosin heavy chain (MyHC) isoforms, are adapted for anaerobic glycolysis and constitute the majority of fish muscle mass. Red muscle fibers, which express slow-twitch MyHC isoforms, are highly aerobic and form a distinct band beneath the lateral line, while pink muscle fibers exhibit intermediate properties and co-express different MyHC isoforms [62]. To examine the higher-order organization within fish muscle tissue, the structure can be considered the connection of repeated segments called myomeres, which are separated by connective tissue sheaths known as myosepta (Figure 1). Each myomere has a three-dimensional W-shaped structure that enables contractions for the efficient bending and flexing of the spine. Locomotion involves the coordinated activity of myomeres and interspersed adipose tissue, with the latter intramuscular fat deposits present between fibers to support energy storage and mechanical function [60].

**Figure 2:**
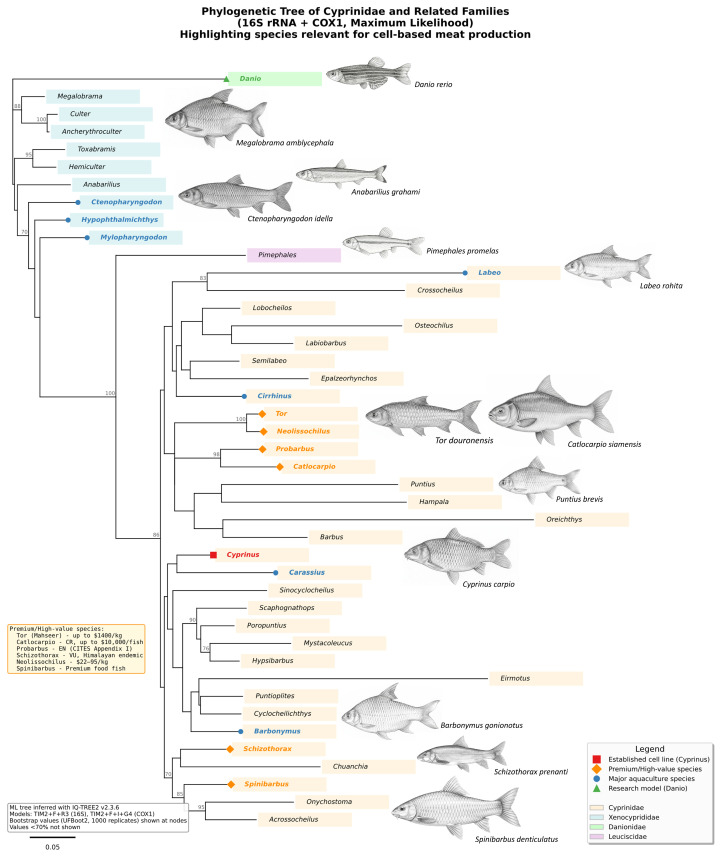
Maximum likelihood phylogenetic tree of Cyprinidae and related families based on concatenated 16S rRNA from 2 datasets and COX1 sequences from NCBI GenBank (1254 bp) [66,67]. The tree was inferred using IQ-TREE v2.3.6 with partition-specific models (TIM2+F+R3 for 16S, TIM2+F+I+G4 for COX1) selected by ModelFinder using BIC [68,69]. Numbers at nodes indicate ultrafast bootstrap support values (1000 replicates) evaluated by SH-like approximate likelihood ratio test (SH-aLRT); values < 70% are not shown [70]. Branch lengths are proportional to the number of nucleotide substitutions per site (scale bar = 0.05). Taxa are colored by family: Cyprinidae (peach), Xenocyprididae (blue), Danionidae (green), and Leuciscidae (purple). Genera with established cell culture systems or major aquaculture importance for cell-based meat production are highlighted: red square = established cell line (*Cyprinus carpio*), orange diamonds = premium/high-value species with potential for cell-based meat production (*Tor douronensis*), blue circles = major aquaculture species, green triangle = research model organism (*Danio rerio*).

These muscle structural and functional characteristics are particularly relevant in the family Cyprinidae, which is currently considered the largest family of freshwater fishes (Class Actinopterygii) and comprises 3006 recognized species across 367 genera [10]. Members of this family include ecologically and economically important species, such as the ornamental Amur carp or koi (*Cyprinus rubrofuscus*), the goldfish (*Carassius auratus*), the common silver barb (*Barbonymus gonionotus*), and the semah mahseer (*Tor douronensis*), which is a valued food fish in Southeast Asia. All of these species contribute substantially to inland fisheries and aquaculture production. In addition to food species, some cyprinids, such as koi and goldfish (*Carassius auratus*), also hold commercial value in the ornamental fish industry. Cyprinids belong to the order *Cypriniformes*. This order also includes related families, such as Danionidae, represented by zebrafish (*Danio rerio*), which is an established vertebrate model organism in developmental and biomedical research [63] (Figure 2). Cyprinidae habitats are remarkably diverse and range from standard freshwater ecosystems to extreme environments, such as high-altitude streams and, in rare cases, brackish waters or those of variable salinity [64].

While recent taxonomic revisions place major aquaculture species like the grass carp (*Ctenopharyngodon idella*) into the sister family Xenocyprididae, both families share exceptional hypoxia tolerance and robust metabolic adaptability, which facilitate scalable bioreactor culture. Nonetheless, the strict Cyprinidae clade offers a unique dual advantage: it encompasses both well-characterized model species that have up to 70 cell lines, including muscle cells and high-value ‘premium’ targets such as *Tor* (Mahseer), *Neolissochilus*, *Catlocarpio* (Giant Barb), and *Spinibarbus*. These species command high market values in Southeast Asian and Indian markets, comparable to marine delicacies, yet they retain the favorable cell-culture attributes of freshwater teleosts [44,45]. This contrasts sharply with high-value marine competitors like Bluefin tuna or Atlantic salmon, which have shown low success in muscle cell culture in terms of isolation yield and post-isolation growth rate [65]. Thus, Cyprinidae provides a strategic ‘sweet spot’: it allows for the development of high-value cultivated products using accessible, scientifically robust biological systems.

## 4. Primary Culture of Fish Muscle Cells

### 4.1. Methods for Establishing Primary Cells

**Figure 3:**
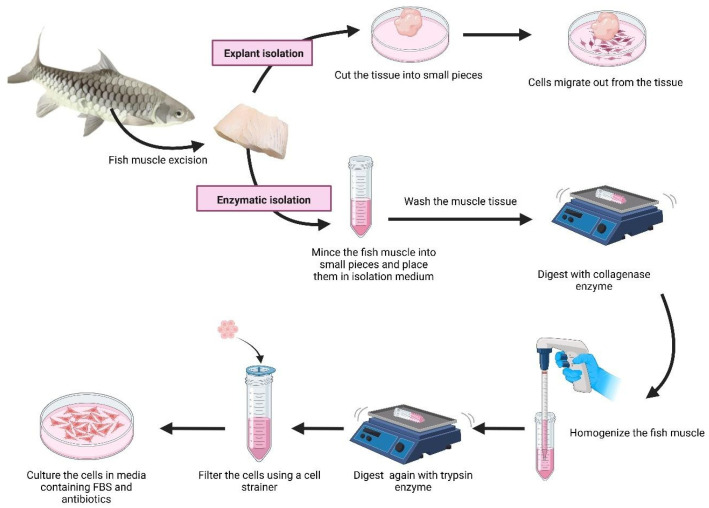
Methods for establishing primary cells using outgrowth and tissue digestion techniques.

There are two principal approaches for establishing primary fish muscle cell cultures: the explant outgrowth method and the tissue digestion or dissociation method (Figure 3). Each technique has specific advantages and limitations, and its suitability is determined based on the species, tissue characteristics, and research objectives.

#### 4.1.1. Explant Outgrowth Method

The explant outgrowth technique is a classic method used to obtain primary cells directly from tissue fragments. In fish, white skeletal muscle is commonly collected from the epaxial region. The tissue is aseptically dissected and cut into small pieces. These fragments are then placed onto a culture surface with an appropriate growth medium [71]. After the cells migrate from the tissue explant, they form a monolayer on the culture plate. When a sufficient number of cells have proliferated, the remaining muscle explant is either removed or transferred to a new culture dish to allow for further outgrowth.

This method is relatively simple, requires minimal equipment, and is cost-effective, which makes it suitable for laboratories with limited resources [72]. However, one major limitation is the heterogeneity of the resulting cell population. Different cell types, such as myoblasts, fibroblasts, and adipocytes, can migrate from the tissue. The resultant mixed cultures may complicate downstream analyses [73]. Furthermore, since the tissue remains exposed to culture conditions for extended periods, there is an increased risk of microbial or fungal contamination, so rigorous aseptic techniques are necessary [74].

#### 4.1.2. Tissue Digestion or Dissociation Method

The tissue digestion method involves the enzymatic dissociation of muscle tissue to obtain a suspension of single cells. Typically, white muscle from the epaxial region above the lateral line is excised and digested using collagenase and/or protease to separate individual cells from the ECM [75]. The resulting cell suspension is then initially filtered through a 100 µm cell strainer to remove debris, with a smaller pore size of around 40 µm used to obtain single cells suitable for culture [76].

This approach generally yields a higher number of viable cells and allows for the enrichment of specific populations, such as muscle satellite cells, through techniques like pre-plating [77]. During pre-plating, fibroblastic cells adhere more rapidly to uncoated or ECM-coated culture surfaces, enabling the selective recovery of less adherent myogenic cells from the supernatant.

Nevertheless, enzymatic digestion can impose harsh mechanical and chemical stress on cells, thereby potentially reducing their viability or damaging the cell surface receptors [78,79]. Proper optimization is species-specific. Careful optimization with respect to enzyme choice (e.g., collagenase, trypsin, dispase), concentration, temperature, and incubation time is therefore critical to improve the cell yield and health.

### 4.2. Culture Conditions

#### 4.2.1. Base Media

Media originally designed for mammalian cell cultures are used for many fish cultures. The factors that are essential for the culture media of fish cells are salt levels, buffer, temperature, sources of carbon, and pH levels [80]. Eagle’s minimal essential medium (MEM) and Leibowitz’s 15 (L-15) medium are the common base media used in fish muscle cell cultures [81].

MEM with fetal bovine serum (FBS) is a favored culture medium suitable for cells from mammals, birds, reptiles, amphibians, and fish [82]. Meanwhile, L-15, a nutrient-rich medium that is high in amino acids and uses a phosphate-based buffer system instead of sodium bicarbonate, does not require a carbon dioxide (CO_2_)-controlled atmosphere [83]. It can maintain pH values within the normal range without additional CO_2_, which allows cells to be grown conveniently at room temperature in any undisturbed area. Because of this advantage, numerous fish cell lines have been successfully established using L-15 media [31,34,84,85,86].

#### 4.2.2. Sera (FBS and Fish Serum)

Animal sera, especially FBS, are widely used in cultures of both mammalian and fish cells. Besides nutrients and pH buffering, FBS contains a variety of growth factors to promote proliferation, signal adhesion to the culture plate, and important soluble proteins, such as metal-ion binding transferrin, which plays a role in iron transport and oxidative stress modulation [87]. Researchers found that increasing the concentration of FBS from 5% to 20% increased the growth rate of muscle cells. However, a concentration of 10–15% FBS resulted in relatively good cell growth, which is advantageous for maintaining cell lines at a lower cost [31,82,85].

Using fish serum or fish muscle extract as substitutes for FBS in fish muscle cultures could be beneficial. According to the findings of a study by Dong et al., fish muscle extract can replace FBS to promote cell proliferation while preserving cell viability and gene expression [31,88]. Replacing FBS is considered a challenge in terms of reducing costs and improving standardization. A defined, serum-free medium for fish muscle cells typically includes a basal medium, such as L-15 or DMEM, and various supplements with various recombinant growth factors, as described in Section 4.2.3 [89].

#### 4.2.3. Supplementation

Basic fibroblast growth factor (bFGF), specifically a human recombinant, is a potent proliferation stimulator that is frequently added to culture media. bFGF plays a crucial role in regulating muscle development and homeostasis and accelerating the regeneration of injured skeletal muscle through the activation of the phosphatidylinositol 3-kinase/protein kinase B/mechanistic target of rapamycin (PI3K/Akt/mTOR) signaling pathway [90]. Despite the inclusion of bFGF in FBS, the concentrations may be insufficient for optimal stem cell proliferation and differentiation prevention. bFGF has been used in several studies to culture myogenic and fibroblast-like cells but not adipogenic cells. bFGF is necessary for the self-renewal of satellite cells as well as for the maintenance and repair of skeletal muscle [91].

Insulin-like growth factor 1 (IGF1) plays a crucial role in protein synthesis and the growth of fish muscle. IGF1 regulates upstream PI3K signaling and is essential for stimulating amino acid metabolism to enhance muscle development. bFGF and IGF1 have the potential to support and enhance the proliferation rate of muscle cell cultures [92]. Other essential components include transferrin (for iron delivery), insulin, selenium, non-essential amino acids, and lipid supplements (especially omega-3 fatty acids). Cost-effective nutrient sources, such as yeast- and plant-based hydrolysates (from soy, peas, and rice), are currently being explored as replacements for expensive recombinant proteins [58].

Epidermal growth factor (EGF) and its receptor (EGFR), a transmembrane glycoprotein with tyrosine kinase activity, constitute a fundamental signaling system regulating cellular proliferation, differentiation, and survival [93]. In teleost fish, the *egfr* gene family has expanded following fish-specific whole genome duplication, resulting in duplicated genes such as *egfra* and *egfrb,* which allow for specialized functions in growth signaling [94]. While the hypothalamic-pituitary-somatotropic axis is the primary endocrine driver of fish growth, the EGF system serves as a vital local regulator [95]. Specifically, EGF binding triggers autophosphorylation of the receptor’s intracellular domain, activating downstream pathways like MAPK/ERK and PI3K/Akt, which are essential for the recruitment and proliferation of myosatellite cells [93,96]. This process is a prerequisite for both hyperplastic and hypertrophic muscle growth, ultimately influencing the muscle cellularity and flesh quality of aquaculture species [96]. In practical applications, studies on teleost species such as the brown-marbled grouper (*Epinephelus fuscoguttatus*) have shown that EGF at a concentration of 20 ng/mL, supplemented with 20 ng/mL bFGF, 20% FBS, and 10% fish muscle extract in L-15 medium, provides the optimal conditions for cell proliferation and the long-term maintenance of stemness across more than 50 passages [97].

#### 4.2.4. ECM

In teleost fish, as in other vertebrates, muscle satellite cells reside in a specialized microenvironment or “niche” located between the sarcolemma of the myofiber and the surrounding basement membrane [98]. This niche is composed of ECM proteins, such as laminin and collagen, which play a critical role in maintaining satellite cell quiescence and regulating their activation, proliferation, and differentiation [99]. Understanding and replicating this niche in vitro is a key goal for successful muscle cell culture. Fish cells may therefore require surfaces that comprise various ECM proteins, such as collagen, fibronectin, polylysine, and laminin, for efficient adhesion and growth. Optimizing these ECM proteins may be critical for efficient fish muscle culture [80,100]. Differences in ECM protein affinity can also influence cell attachment behavior and thereby support selective enrichment strategies during primary muscle cell culture. The difference in ECM protein affinity would also assist cell purification, since the initial cell isolate is a heterogeneous population [99]. Both the cyprinid clade and Atlantic salmon primary muscle cells have been successfully cultured and differentiated on laminin-coated tissue culture plates [100,101]. Consequently, research into the ECM required for fish cell survival and proliferation in vitro is a significant area of investigation.

#### 4.2.5. Temperature

Fish cell cultures have several differences compared to mammalian cell cultures. These include their ability to adapt to a wide range of temperatures and a higher tolerance to hypoxia [102]. As fish are ectothermic animals, the optimal temperatures for fish cell cultures may vary depending on the natural habitats of the fish, which may vary from 15 °C to 30 °C. The primary muscle cells of trout and sea bream were maintained at temperatures of 15–21 °C [103].

Culturing fish muscle cells at lower temperatures can reduce the energy required to maintain controlled culture conditions. This reduction in energy demand may translate into cost savings during large-scale production of cell-based aquatic foods. Compared with cell-based terrestrial meats, cell-based aquatic foods therefore have the potential to offer economic advantages at an industrial scale [80].

#### 4.2.6. CO_2_

While mammalian cells need 5% CO_2_ to maintain a pH of around 7.4 via a bicarbonate buffer system catalyzed by carbonic anhydrase, excessive levels of CO_2_ in the aquatic environments of aquaculture can be detrimental to fish. When the concentration of CO_2_ surpasses the safe threshold, it significantly reduces the capacity of fish blood hemoglobin to carry oxygen, leading to results in fish experiencing respiratory distress [104]. This can happen even when the water contains high concentrations of dissolved oxygen. However, fish cells are well adapted to pH variability and do not need CO_2_ regulation. Nonetheless, fish cells still require culture media with a pH buffer quality. L-15 with HEPES for extra buffering capacity is therefore widely used as a culture medium for fish cells [80].

## 5. Characterization of Cell Culture Systems

### 5.1. Morphology

Muscle tissue contains two primary cell types that are capable of expansion and differentiation into muscle fibers, namely, satellite cells and myoblasts. Satellite cells function as muscle stem cells, are typically small and flattened, and reside between the sarcolemma and basal lamina [83]. In primary culture, a satellite cell generally assumes a spindle shape. Given their metabolically quiescent state, satellite cells exhibit a high nucleus-to-cytoplasm ratio, with a densely packed, heterochromatic nucleus and few organelles aside from ribosomes, which are required to synthesize the proteins essential for myogenesis [105].

In response to stress or muscle injury, satellite cells are activated to proliferate and differentiate into myoblasts. Myoblasts are larger and more round or oval than satellite cells. A larger cytoplasm ratio and more organelles reflect their increased metabolism. Myoblasts retain a proliferative potential and ultimately fuse to form multinucleated myotubes, which mature into functional muscle fibers. Importantly, myoblasts are differentiated from satellite cells primarily by their active participation in muscle growth and repair rather than by their distinct morphological characteristics [106].

Another key cell type within muscle tissue is the fibroblast. Fibroblast-like cells can be derived from multiple sources, including muscle tissue and the caudal fin. Proliferative fibroblasts are typically elongated and spindle-shaped [107]. When ready to produce ECM, fibroblasts assume more extensive cytoplasmic branching and accumulate a rough endoplasmic reticulum. While mature fibroblasts and their ECM products are essential to muscle development and regeneration by satellite cells, an excessive number of mature fibroblasts or myofibroblasts may cause fibrosis and subsequent muscle dysfunction [108].

Primary preadipocytes are proliferative precursors to lipid-storing adipocytes. These cells exhibit a fibroblast-like morphology characterized by a small, thin cytoplasm devoid of lipid droplets. When isolated from the adipose tissue of grass carp (*Ctenopharyngodon idella*) and cultured with 10% FBS at 28 °C in a humidified 5% CO_2_ atmosphere, they reach confluence within 10 days. They subsequently gradually accumulate lipid droplets and adopt a rounder morphology until they become fully differentiated by day 20 [6]. The optimal culture temperature for preadipocytes varies among fish species, thus reflecting their native habitats. For example, common carp preadipocytes are cultured at 23 °C and those of Atlantic salmon at 12 °C [109,110].

Cellular senescence is a state of stable cell cycle arrest, in which cells remain viable and metabolically active but lose the ability to divide. This poses a significant challenge for the development of cell lines, which require the capacity for continuous proliferation. Senescent cells are typically characterized by flattened and enlarged cell bodies with heterochromatic nuclei. While satellite cells may also exhibit a flattened morphology within their physiological niche, they generally adopt a compact spindle shape in 2D culture systems. Nevertheless, unbiased morphological assessments of senescence across large cell populations remain challenging using conventional methods. To address this, deep learning models have been developed to facilitate senescence detection [111,112]. Recent models have shown that senescence can be accurately inferred from nuclear morphology alone, with features such as a high aspect ratio and low convexity serving as reliable markers [112].

Differentiation via induction, which is typically achieved by reducing the growth factors in the medium, can be assessed by applying distinct methods. The observed ability of myoblasts to fuse into multinucleated myotubes can be determined using the fusion index, which is defined as the percentage of total nuclei within MyHC-positive myotubes (see Section 5.2 for more on MyHC). Morphological inspections can also be performed concurrently with contractility assessments [113,114]. The latter can be conducted either by observing spontaneous contractions or those induced in response to electrical stimulation. The use of microscopes allows karyotyping (analysis of the chromosome number and structure) and the examination of genetic stability. Since long-term culture can lead to chromosomal abnormalities and genetic drift, periodic karyotyping is essential to ensure the genetic stability of cell lines, as such stability is critical for both research reproducibility and food safety.

### 5.2. Molecular Characterization in Fish Muscle Cell Cultures

Satellite cells and myoblasts, which are key progenitors in muscle development, are marked by at least Paired Box 7 (Pax7), a paired-box transcription factor protein, and Myogenic Differentiation 1 (MyoD), a member of the myogenic regulatory factor (MRF) family. Pax7, the expression of which is typically downregulated as cells commit to terminal differentiation, is the definitive marker for quiescent and proliferating satellite cells [115]. In *Cyprinus carpio*, the primary satellite cells exhibit a brief proliferative phase before differentiating and fusing into myotubes, largely independent of the tissue source or media composition. In marine species, such as *Sebastes schlegelii*, Pax7-positive satellite cells rapidly differentiate into myoblasts within five passages, with the resulting myoblast pool capable of stable expansion for over 50 passages [116]. Some fish species also express a related factor, Pax3 [117], in muscle progenitors; zebrafish have Pax3a/Pax3b genes whose expression overlaps with Pax7 in early myogenesis. Both Pax7 and Pax3 are considered conserved markers of the muscle stem cell lineage across vertebrates [118]. Satellite cells can be identified by distinctive cell-surface proteins. Notably, the receptor for hepatocyte growth factor c-Met (also known as Met) and the adhesion molecule M-cadherin (Cadherin-15) are classic satellite cell markers in mammals, and fish satellite cells appear to share these features [119]. Another surface marker is Syndecan-4, a cell membrane proteoglycan, expressed in fish muscle satellite cell populations [117]. Mammalian satellite cells are also known to express CD34 (a stem cell surface marker) and VCAM-1 (vascular cell adhesion molecule) on their surface, and while less documented in fish, the conserved presence of such receptors in the fish myogenic satellite cells is likely. These surface markers are important because they would enable the isolation and purification of muscle stem cells by fluorescence-activated cell sorting (FACS) or magnetic sorting in principle. Indeed, combinations like Pax7^+^/c-Met^+^ or Syndecan-4^+^ could be applied for the fish muscle progenitors.

**Figure 4:**
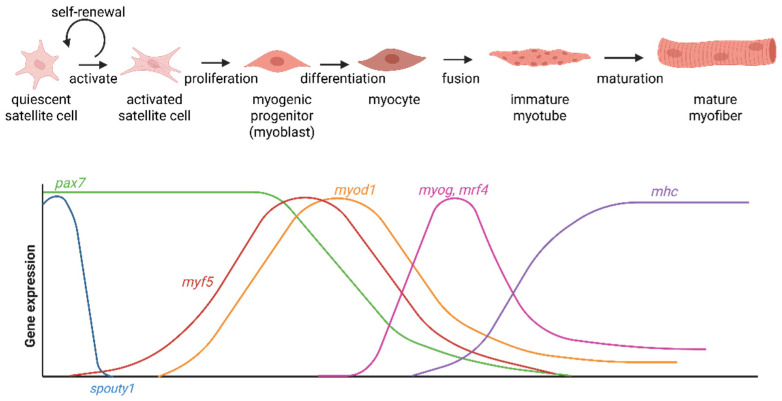
Schematic of fish skeletal muscle development and stage-specific myogenic markers. This diagram illustrates fish myogenesis, in which Pax7^+^ satellite cells become activated and proliferate into Myf5^+^/MyoD^+^ myoblasts. These cells subsequently differentiate into myogenin^+^ myotubes before maturing into functional myofibers that express structural markers such as MyHC and Mylz2.

In myoblasts that have committed to the myogenic lineage, MyoD expression increases during proliferation and early differentiation [120]. MyoD expression fluctuates during differentiation but generally decreases in myotubes. As shown in Figure 4, typically, an activated progenitor will upregulate MyoD while Pax7 levels decline, indicating that the cell is leaving the stem state and becoming a committed myoblast. MyoD is a conserved master regulator of myogenic fate, and its expression is a hallmark of myoblast identity in fish and mammals alike. Other intracellular markers of myoblasts include Myf5 (myogenic factor 5, another early MRF, which often initiates slightly earlier than MyoD) and desmin, an intermediate filament protein [121,122]. Desmin is a muscle-specific structural protein; in mammals, activated satellite cells begin expressing desmin as they differentiate. In zebrafish primary muscle cell cultures and spotted skat cell cultures, proliferating muscle progenitors have indeed been reported as desmin-positive, underscoring that the myogenic lineage is maintained [42]. Myogenin (MyoG), a helix–loop–helix transcription factor specifically for muscle fiber differentiation (another MRF), increases from the onset of terminal differentiation and peaks during myoblast fusion or functional myotube formation. MyHC is upregulated only after functional maturation, thereby contributing directly to muscle contraction and acting as a marker for mature, fused myotubes. MyHC is expressed only in terminally differentiated muscle cells [123] (Figure 4). For example, in cultured Atlantic mackerel (a teleost fish) muscle cells, researchers confirmed differentiation by the presence of MHC in multinucleated cells [65]. Similarly, Troponin proteins (such as Troponin T and I, components of the thin filament regulatory complex) are upregulated in differentiating fish muscle; the study of mackerel cells also tracked TNNT3a (troponin T isoform) as a differentiation gene that increases alongside myogenin [65]. Mammalian myoblasts (derived from activated satellite cells) are known to express NCAM (neural cell adhesion molecule, also known as CD56), and retain M-cadherin and c-met [124]. It is likely that fish myoblasts share some of the adhesive proteins for surface markers as seen in other vertebrates.

Differentiation can be monitored using multiple other markers. Ki67, which is expressed during all active cell cycle phases (G1, S, G2, M), declines sharply as myoblasts exit the cycle. Proliferating cell nuclear antigen (PCNA) persists longer and may remain detectable in early differentiated cells. Cell proliferation is antagonistically regulated with differentiation and apoptosis: PI3K/Akt-associated β1-integrin decreases during differentiation; however, caspase-9 increases, with a peak around day 2 and a return to baseline upon functional myotube formation. In *Labeo rohita* myogenic cell lines, satellite cells dominate the initial population, while MyoD-positive myoblasts are the main proliferative population by passage 15. Later passages (≥25) show increased expression of MEF2A, Mrf-4, and MyoG, which reflects mixed populations of differentiated myotubes and mitotic myoblasts, although detailed cell subtype profiling has not been conducted to date [45]. Notably, zebrafish muscle stem cells exhibit peak MyoD expression during early myotube formation rather than in the myoblast phase, likely due to the gradual accumulation of MyoD mRNA during differentiation [42].

These markers can therefore be used for immunocytochemistry characterization, such as immunofluorescence, and allow for the quantification of gene expression using real-time polymerase chain reaction (RT-PCR) throughout the culture and differentiation process as measurements of myogenic progression [125,126].

In addition to myogenic composition, the muscle consists of fibroblasts and some adipocytes. Fibroblast-like cells are common in established Cyprinid cell lines and are characterized by vimentin expression. Tor caudal fin fibroblasts express vimentin but not cytokeratin [35]. In marine fish, turbot (*Scophthalmus maximus* L.) muscle fibroblasts express TCF-4, a signaling factor that promotes myogenesis, but lack Pax7 and desmin [127]. Preadipocytes, which are proliferative precursors of lipid-storing adipocytes, maintain a fibroblast-like morphology before confluence. Differentiation is accompanied by increased glycerol-3-phosphate dehydrogenase activity and the upregulation of peroxisome proliferator-activated receptor gamma (PPAR-γ), with lipid droplet formation visualized via Oil Red O staining [128].

## 6. Applications of Cultivated Fish Meat and Future Perspectives

### 6.1. End-Product Landscape: From Unstructured Biomass to Final Consumer Goods

The ultimate goal of cultivated fish production is to deliver safe, nutritious, and appealing products to consumers. The transition from cellular biomass to a final product involves sophisticated multi-disciplinary fields, such as bioprocess and tissue engineering and food science, to replicate the complex sensory attributes of conventional aquatic food. The initial output from bioreactors is typically an unstructured slurry of muscle (or myogenic lineage) cells, which can be readily used for products where the original muscle structure is not critical, such as surimi, fish balls, fish cakes, dumplings, or fillings [80,129]. However, to replicate high-value conventional aquatic food, the industry is focused on creating structured products like fillets and steaks.

Achieving the characteristic texture, flavor, and appearance of a fish fillet requires the co-culturing of muscle, fat, and connective tissue cells on edible scaffolds that guide their organization into aligned fibers [75,130]. The composition and distribution of intramuscular fat, for instance, are critical for delivering the desired juiciness, mouthfeel, and species-specific flavor profile. Furthermore, hybrid products, which combine cultivated fish cells with plant-based or fungal-derived proteins and fats, could represent a pragmatic near-term strategy. This approach can significantly improve texture, reduce production costs, and accelerate market entry while still delivering an authentic aquatic food experience that is superior to purely plant-based alternatives [131]. Companies like BlueNalu and Finless Foods are actively developing both fully cultivated and hybrid products, targeting high-demand species such as bluefin tuna and salmon, where the environmental impacts—conservation of the species’ natural habitats, and supply chain benefits—no longer require transporting fish over long distances—are most pronounced [132].

### 6.2. Market Integration: Consumer Acceptance, Regulatory Pathways, and Commercial Viability

The successful application of cultivated fish meat relies on three interconnected factors: consumer acceptance, a clear regulatory pathway, and commercial viability. First, consumer interest is driven by a growing awareness of the environmental unsustainability of conventional fishing [129], including overfishing, habitat destruction, and bycatch, as well as concerns about contaminants like heavy metals [133] and microplastics [134] found in wild-caught fish. Cultivated aquatic food offers a compelling solution, providing a clean, safe, and ethically produced protein source with a significantly lower environmental footprint [80]. However, consumer neophobia, price sensitivity, and “unnaturalness” perceptions remain significant obstacles to widespread and mass adoption [135]. Overcoming these barriers will require transparent communication, educational marketing, and achieving comparable pricing with conventional aquatic food.

Second, the regulatory landscape is also a critical factor for market entry. Singapore was the first to approve the sale of a cultivated meat product in 2020 [136]. The United States followed with its first approvals in 2023, establishing a dual-agency framework between the FDA and USDA for safety, production and labelling cultivated meat, while the FDA is solely responsible for cultivated seafood [137]. This progress provides a roadmap for other nations, but navigating the regulatory requirements in major markets like the European Union remains a complex and time-consuming process. As of 2024, the cultivated seafood sector includes over 25 companies globally, signaling strong commercial interest, but achieving the scale and cost-efficiency required for mass-market viability remains a primary long-term challenge [132]. Intertwined with these two described factors, the viable commercialization of cell-based aquatic food would still hinge on the foundational technologies for its mass production elaborated in the subsequent section.

### 6.3. Enabling Technologies for Cultivated Fish Meat Production Beyond Cell Line Development

Cell-based fish systems represent a promising approach to the production of animal-free aquatic foods. By combining advances in biomedical engineering and aquaculture, these systems enable the cultivation of marine animal cells at scale [138]. The techniques developed for mammalian cell production, such as closed-system bioreactors, can be adapted for large-scale fish cell culture. Compared to mammalian muscle, fish muscle tissue is particularly amenable to bioreactor cultivation owing to its physiological characteristics [28]. Cyprinids are attractive targets for cell-based meat due to their abundant intermuscular bones, which pose health risks and reduce consumer appeal [139,140]. Fish cells also exhibit enhanced hypoxia tolerance, which reduces the need for intensive oxygenation in bioreactors. Furthermore, their broad pH tolerance allows growth across a wider pH range, and their ability to thrive at lower temperatures reduces their heat transfer requirements and overall production costs [141].

#### 6.3.1. Scaffolds for Three-Dimensional Tissue Cultivation

In fish cell culture, the process of cultivating three-dimensional tissues is dependent on the presence of scaffolds. Scaffolds are biocompatible materials that serve as a support system for the growth and differentiation of fish cells by providing an ideal morphology, chemical composition, and structural template [142]. They play a crucial role in mimicking the natural microenvironment of fish tissues, thereby facilitating the formation of functional tissue structures. They provide physical support to the cells, which allows them to organize and interact with one another in a manner similar to their natural counterparts [143]. Scaffolds can also influence cellular behavior and promote cell adhesion, proliferation, and differentiation [144].

Several types of scaffolding materials used in medical tissue engineering, such as cellulose, alginate, and chitosan, can be applied in fish cell culture [144]. Cellulose, a polysaccharide found in plant cell walls, is known for its biocompatibility and mechanical strength. Alginate, which is sourced from seaweed, possesses excellent gel-forming properties and can encapsulate fish cells for further cultivation [145]. Chitosan, derived from the exoskeleton of crustaceans, exhibits antibacterial properties and can promote cell adhesion and growth [141]. Beyond cellulose, alginate, and chitosan, decellularized ECM scaffolds derived from plant tissues (e.g., spinach, apple) and fungal mycelium are emerging as promising, edible scaffolds. These materials can provide a pre-existing vascular-like network and natural structural support. By employing suitable scaffolds in fish cell culture, researchers aim to create environments that support the growth and development of fish tissues in vitro to ultimately advance the field of cultivated fish products and contribute to sustainable aquatic foods production [80,146].

#### 6.3.2. Bioreactors

The success of cell-based aquaculture depends on several key factors, including the development of suitable muscle cell sources, the optimization of culture conditions, and the ability to achieve large-scale cell production in bioreactors [147]. Bioreactors provide a controlled and optimized environment for cell growth by regulating parameters such as pH, temperature, and oxygen concentration [148]. A large surface area is required to generate sufficient numbers of muscle cells for downstream applications [149]. In vitro meat production, therefore, necessitates the use of large-capacity bioreactors capable of supporting the expansion of stem cells and differentiated skeletal muscle cells. Importantly, the bioreactor environment must closely mimic the physiological conditions found in vivo, particularly with respect to temperature and oxygen levels. Cell cultivation can be performed using either fed-batch or continuous culture systems [150,151].

The most commonly used bioreactor types in cell-based aquaculture and cultured meat production are stirred-tank and fixed-bed bioreactors [138]. In large-scale production, stirred-tank bioreactors are well suited for suspended cell cultures, but they can also be adapted for adherent cells through the use of microcarriers (i.e., small beads that are generally 100–300 µm in diameter), which provide a large surface area for attachment. The agitation speed needs to be carefully controlled to ensure adequate nutrient mixing and oxygen transfer without causing excessive shear stress on the cells [152]. In contrast, fixed-bed bioreactors are typically employed for adherent cell types. These systems contain stationary scaffolds (e.g., packed or hollow fiber matrix materials) that provide a large surface area for cell attachment and growth, and the culture medium is perfused through the bed [149,153]. This configuration can reduce shear stress and is particularly suitable for cells that are sensitive to mechanical forces. Nonetheless, scaling up from laboratory flasks to industrial-scale bioreactors presents significant challenges. Maintaining homogeneity in large volumes, ensuring sterility, managing heat transfer, and controlling critical process parameters (i.e., pH, dissolved oxygen, glucose, lactate) all become more complex. The implementation of process analytical technology, which involves real-time monitoring and feedback control, is essential for achieving consistent product quality at scale [154].

#### 6.3.3. Advanced Cellular Characterization Using Single-Cell Transcriptomics for Optimizing Piscine Myogenic Cultures

While traditional marker analysis provides valuable insights, the advent of single-cell RNA sequencing (scRNA-seq) has revolutionized the understanding of cellular heterogeneity within muscle tissue by enabling high-resolution transcriptomic profiling of individual cells [155]. This technology overcomes the limitations of bulk RNA sequencing, which provides only an averaged view of gene expression, and has been instrumental in dissecting the complex cellular landscape of myogenesis. scRNA-seq studies have revealed that the satellite cell pool, once thought to be relatively homogeneous, is composed of multiple subpopulations with distinct transcriptional signatures and functional potentials [156,157]. For instance, scRNA-seq has been used to identify functionally heterogeneous human satellite cells and to characterize a reference atlas of human skeletal muscle tissue, revealing bifurcated muscle stem cell populations [158,159].

Furthermore, scRNA-seq allows for the reconstruction of developmental trajectories, providing a dynamic view of the transitions from quiescent satellite cells to activated myoblasts and their subsequent differentiation into myotubes. This has led to the identification of transient cellular states and novel regulatory networks that govern cell fate decisions during myogenesis [160]. The application of scRNA-seq to aquaculture species holds significant promise for advancing our understanding of fish muscle development and for optimizing cell-based meat production. By characterizing the cellular heterogeneity of fish muscle cell cultures, scRNA-seq can facilitate the identification of superior cell lines, the development of more effective differentiation protocols, and the discovery of novel biomarkers for monitoring cell state and quality. The high-resolution data provides a transcriptomic roadmap to rationally design culture conditions and select for cell populations with high-performing myogenic potential, which is essential for optimizing the efficiency, scalability, and consistency of piscine muscle production.

## 7. Limitations

Despite substantial progress, several key challenges remain for the cultivated meat industry, particularly in the development of cell-based aquatic foods [81]. Although multiple protocols have been reported for the isolation and culture of fish muscle cells, the lack of standardized criteria for defining myogenic fish cultures remains a persistent challenge in cell-based meat research. The wide variability in tissue source, isolation methods, culture media, and differentiation conditions limits reproducibility and meaningful comparison across studies. Therefore, there is a clear need to establish standardized criteria to define and validate myogenic fish cultures. Such criteria should include confirmation of myogenic identity through conserved marker expression, including Pax7 for satellite cells, MyoD, Myf5, or desmin for proliferating myoblasts, and myogenin and myosin heavy chain for differentiated myotubes [115,120,123]. In addition, quantitative assessment of fusion capacity and myotube formation, as well as systematic evaluation of fibroblast contamination using morphological characteristics and molecular markers such as vimentin, should be routinely performed [35]. Long-term passage stability, including proliferative capacity, maintenance of myogenic marker expression, and chromosomal integrity, should also be evaluated over extended culture [161]. The adoption of standardized definitions and evaluation criteria would improve reproducibility, facilitate cross-study comparisons, and support the development of scalable cultivated fish meat systems.

### 7.1. Limited Aquatic Foods Cell Lines

Only a limited number of established fish muscle cell lines are currently available, which poses a major obstacle to the large-scale production of cell-based aquatic foods. In the absence of commercially available fish muscle cell lines, researchers must rely on freshly obtained fish specimens to isolate primary muscle cells. Moreover, the lack of optimized culture conditions for many fish species has limited the successful establishment and maintenance of stable fish cell lines [97]. To promote the broader application of fish cell lines as standard research tools, standardized methodologies, including the use of defined media and reagents, consistent equipment, and rigorous quality control protocols, need to be implemented. The proper characterization and detailed documentation of newly developed cell lines would further enhance reproducibility, accelerate progress, and expand research potential in aquatic cell culture [1,80].

### 7.2. Lack of Effective Serum-Free Media

One of the major challenges in cultivated meat production is the development of suitable serum-free culture media. Alternatives that optimize the composition of media while lowering costs and reducing dependence on animal-derived components have not been fully explored [162]. Currently, FBS is widely used, but it introduces several limitations, including high costs, potential contamination, supply instability, batch variability, ethical issues, and downstream processing difficulties [163]. To date, no fish cell lines have been reported that can sustain robust proliferation under completely serum-free conditions. Addressing this limitation requires focused efforts to optimize culture formulations and identify affordable, serum-free alternatives, which will ultimately promote the cost-effective and ethically sustainable production of cultivated fish meat [81,132].

### 7.3. High Production Costs

Reducing production costs to compete with conventional piscine meat remains a primary goal for the commercialization of cell-based fish. Techno-economic analyses have indicated that the culture medium is the single largest cost driver, as it can account for over 50% of the total cost of unoptimized processes [164]. The high prices of recombinant growth factors (especially bFGF, which can cost thousands of dollars per gram) and FBS are the main contributors [25].

Evaluating the scalability of cultivated fish meat requires a thorough analysis of production yield and efficiency, particularly in comparison to more established terrestrial cultured meat systems. For terrestrial cultured meat, significant progress has been made in reducing production costs—from the initial multi-million-dollar burger to current techno-economic targets of approximately $63/kg [165]. The projected figure was derived from assumptions related to growth medium, bioreactors, equipment, and labor under many best-case scenarios—e.g., the successful development and commercialization of serum-free media and a reduction in growth medium costs to $374/L. These advancements are closely tied to improvements in key production metrics, such as achieving high cell densities in large-scale bioreactors (e.g., 10–20 m^3^) and optimizing the metabolic efficiency of cell lines [164]. The inherent physiological advantages of cell-based fish could yield superior production efficiencies to that of terrestrial animals, such as lower culture temperature, reducing energy to heat the bioreactors, hypoxia tolerance and wider pH range, simplifying the bioreactor designs and supporting higher cell density [80]. Forsea, a cell-based fish company, reported cell densities exceeding 300 million cells/mL for eel cells, a benchmark that is substantially higher than what is typically achieved in terrestrial cell cultures [166].

Therefore, future research should focus on innovating affordable media components (e.g., low-cost growth factor alternatives) and scalable formulations that can maintain cell growth, differentiation, and product quality. Implementing cost-effective bioprocess strategies and developing large-scale production systems will also be essential to achieving the target production cost of $63/kg or less, which will make cultivated fish meat economically viable and a competitive alternative to conventional aquaculture and wild-caught seafood [165,167]. Cultivated fish meat is unlikely to supplant conventional aquaculture or the employment it supports; rather, it is expected to serve as a complementary source that helps meet anticipated growth in future consumption while alleviating pressure on wild fisheries.

## 8. Outlook

The transition of cultivated fish meat from laboratory research to industrial scale depends on the strategic selection of species, cell sources, and cultivation methodologies. Among the diverse taxa studied, fish in the Cyprinidae family, particularly *Carassius auratus* (goldfish) and *Cyprinus carpio* (common carp), emerge as the most promising candidates for large-scale production due to their biological resilience and established genomic data. Recent breakthroughs have demonstrated the successful preparation of centimeter-scale cultivated meat from *C. auratus* using scalable expansion techniques [168]. Additionally, high-value species such as the Japanese eel (*Anguilla japonica*) and Large yellow croaker (*Larimichthys crocea*) are highly promising due to the establishment of stable, spontaneously immortalized myoblast cell lines, which ensure consistency in long-term manufacturing [169,170]. This technological framework provides a robust foundation for developing cultivated products from high-value local Cyprinids, such as those found in Southeast Asia, which could significantly impact regional prosperity. Regarding the tissue source, satellite cells derived from epaxial skeletal muscle remain the gold standard. These cells exhibit superior myogenic potential and robust proliferation rates, which are essential for generating dense muscle tissue [171]. However, as observed in our review of primary cultures, maintaining cell purity throughout successive passages is a critical challenge. The encroachment of fibroblast-like cells can diminish the myogenic quality of the final product, necessitating rigorous selective enrichment or single-cell cloning strategies to maintain lineage integrity.

The most effective culture strategies involve a transition toward serum-free media supplemented with specific growth enhancers like hydrocortisone, bFGF, and IGF-1 to ensure food safety and cost-efficiency [170]. For structural development, the integration of 3D edible porous microcarriers, often composed of natural polymers like gelatin or chitosan, within bioreactor systems is currently the most viable approach for achieving mass production [168]. Furthermore, innovative co-culture systems or the use of versatile cell populations (e.g., fibroblasts capable of both myogenic and adipogenic differentiation) represent a significant frontier. These strategies aim to replicate the complex lipid profiles and textural integrity of conventional fish fillets [172].

Beyond the core challenges of cell line availability and culture media costs, several additional factors will be critical to the future success of cultivated fish meat. One key factor is genetic and epigenetic stability. Long-term cell culture can lead to genetic drift, chromosomal abnormalities, and cellular senescence (the Hayflick limit). Therefore, routine karyotyping and monitoring of telomerase activity at predetermined passages will be necessary to ensure the stability and safety of cell lines used for food production. In some cases, controlled immortalization strategies may be required to extend the replicative lifespan of cells; however, these approaches must be carefully evaluated for safety.

Sensory and nutritional fidelity should also be considered. Replicating the complex texture and flavor of conventional fish meat remains a major challenge. This process involves not only the culture of muscle cells but also their co-culture with adipocytes to achieve the desired marbling and lipid profile, particularly the beneficial omega-3 fatty acids, including eicosapentaenoic acid (EPA) and docosahexaenoic acid (DHA), which are hallmarks of fish nutrition. The texture of the final product depends on the alignment and maturation of muscle fibers, which can be influenced by mechanical stimulation and electrical pacing during culture. While early products may be unstructured (e.g., minced fish for fish cakes or sausages), the production of structured products, such as fillets, will require advanced edible scaffolds to guide three-dimensional cell organization. Ideally, these scaffolds should be derived from sustainable sources, such as plants or algae, and should degrade or integrate into the final product. Finally, successful commercialization will depend on navigating regulatory frameworks and gaining consumer trust. Clear labeling, transparent communication regarding the production process, and rigorous safety testing will be essential. Acceptance by future generations of consumers is likely to increase if the environmental and ethical benefits of cultivated fish meat are clearly communicated.

## 9. Conclusions

The reviewed literature indicates that cyprinid species show strong potential for cultivated fish meat production. White skeletal muscle from the epaxial region emerges as the most suitable tissue source due to its enrichment in myogenic progenitors and direct relevance to edible muscle formation. Culture strategies integrating enzymatic dissociation, selective enrichment, and supplementation with myogenic growth factors (e.g., bFGF, IGF-1, and EGF) appear most effective for expanding myogenic populations while supporting differentiation.

Cultivated fish meat offers a promising avenue for sustainable protein production through the integration of aquaculture, biotechnology, and food security. Species within the Cyprinidae family, supported by advances in primary culture techniques, optimized growth conditions, and emerging technologies, such as scaffolds and bioreactors, provide a valuable foundation for the development of muscle cell culture systems. However, progress remains constrained by the limited availability of standardized cell lines, a dependence on serum-based media, and the high cost of large-scale production. Addressing these challenges will require coordinated efforts across the fields of cell biology, materials science, and bioengineering. With continued innovation, cell-based fish products could reduce reliance on wild fisheries and provide safe, sustainable, and accessible protein for human consumption into the future.

## Data Availability

No new data were created or analyzed in this study. Data sharing is not applicable to this article.
